# Urgent and Nonurgent Presentations to a Psychiatric Emergency Service in Nigeria: Pattern and Correlates

**DOI:** 10.1155/2014/479081

**Published:** 2014-01-30

**Authors:** Increase Ibukun Adeosun, Abosede Adekeji Adegbohun, Oyetayo Oyewunmi Jeje, Olufemi Oyeleke Oyekunle, Modupeola Olugbemisola Omoniyi

**Affiliations:** Federal Neuro-Psychiatric Hospital Yaba, 8 Harvey Road, PMB 2008, Lagos, Nigeria

## Abstract

Psychiatric emergencies are acute mental health disturbances that require immediate intervention. However, the emergency department is increasingly being utilised for nonurgent mental health problems, thereby compromising the quality of care available for patients with urgent problems. This study assessed the level and correlates of urgency of mental health problems among patients presenting to an emergency department in Nigeria. The Crisis Triage Rating Scale, Clinical Global Impression Scale and a supplementary questionnaire were administered to 700 attendees at the emergency department of the Federal Neuro-Psychiatric Hospital Yaba, Lagos. Only 29.1% of the presentations constituted an “emergency” 10.9% were “urgent,” while 60% were “nonurgent.” The most common reason for nonurgent presentations was the need for medication refill. On regression analysis, level of urgency of presentations was independently associated with employment status, need for medication refill, substance abuse, suicidality, routine clinic attendance, and use of physical restraint before presentation. The majority of visits to the emergency department are for apparently “nonurgent problems.” However in a resource-poor setting, the emergency department may be the only safety net for the attendees. Our findings point to a need for education of service users and policy shifts in mental health care financing and organisation.

## 1. Introduction

By definition, psychiatric emergencies are acute disturbances of thought, mood, behavior, or social relationships that require immediate interventions [1]. Psychiatric emergencies may also be defined as circumstances capable of resulting in a catastrophic outcome without the availability of resources to deal with the situation at the time and place of occurrence [2]. On the other hand, nonurgent conditions evolve more slowly, the feared outcome is not imminent, and interventions can be sought in routine mental health care facilities such as outpatient clinics [1].

The major role of Psychiatric Emergency Services (PES) is to cater for patients with acute mental health problems or crisis, after which the patients are discharged to continue treatment in routine mental health settings. However, studies have demonstrated a tremendous increase in the number of visits to PES and a trend towards the utilisation of the PES as the sole point of care for patients with nonurgent problems or those whose presentation could have been avoided by adherence with routine outpatient care [3–9].

The oversubscription of the PES by patients without urgent problems exerts constraint on the resources available to cater for patients in acute crisis and leads to overcrowding of the emergency department, thereby compromising the delivery of timely efficient interventions for patients who require emergency attention [3]. It is also costly, imposing a huge financial burden on health systems. Efficient triaging, which is hinged on the differentiation between urgent and nonurgent presentations, has been recognised as a quality indicator of care standards in Psychiatric Emergency Services, because it facilitates the matching of acuity of problems with appropriate level of intervention. However, presentations to PES may be inevitable when there are several barriers to accessing routine mental health services [4].

Previous studies conducted in western countries found that ethnic minorities, immigrants, and patients with low socioeconomic status and poor social support are more likely to use PES excessively for nonurgent problems [10]. Defaulting or dropping out from out-patient treatment, poor access to routine mental health care, and other unmet needs for mental health services may also account for the overutilisation of PES for nonacute problems [11].

There is a research gap on the urgency of mental health problems among patients presenting to PES in sub-Saharan Africa. Differences in health system structure, health care financing, and sociocultural contexts may limit the applicability of the findings of studies conducted in developed countries to a sub-Saharan African setting. Data on the urgency of presentations to our PES will inform the planning of health resources, policies, and interventions directed towards the matching of patients's needs with resource provision. It may also serve as proxy indicator of the functioning and connectedness of the other components of the mental health system of care delivery. Therefore, the current study aimed to assess the levels of urgency (emergency, urgent, nonurgent) of mental health problems among patients attending a psychiatric emergency department in Lagos, Nigeria. The factors associated with the urgency of problems at presentations were also determined.

## 2. Materials and Methods

### 2.1. Study Setting

The study was conducted at the largest mental health care facility in Nigeria, Federal Neuro-Psychiatric Hospital Yaba, Lagos. The Emergency Unit of the Federal Neuro-Psychiatric Hospital Yaba is the only facility-based 24-hour Psychiatric Emergency Service with on-site consultant psychiatrist in Lagos, a metropolis with a population of about 10 million. The Emergency Unit is also staffed with psychiatry residents, psychiatric nurses, social workers, crises staff and other paramedical/auxiliary staff. The facility has an open-door policy and no patient is turned back on account of being a “nonurgent” presentation.

### 2.2. Subjects

Consecutively presenting patients (*n* = 700) to the emergency department of the hospital, with primary diagnosis of a psychiatric disorder, according to the International Classification of Diseases (ICD-10) criteria [12], were recruited into the study between July and September, 2012. Patients with primary medical/neurological disorders who needed to be referred for acute care were excluded.

### 2.3. Procedure

Approval was obtained from the Research and Ethical Committee of the Institution. The study used a prospective design. All patients with a primary diagnosis of psychiatric disorder who met the inclusion criteria were consecutively recruited as part of routine clinical procedures after obtaining their consent. Data were obtained through face-to-face interview with patients and accompanying persons, conducted by the consultant psychiatrist and senior psychiatry residents. The Crisis Triage Rating Scale [13] was administered during the initial assessment, while other measures were completed by the attending clinicians after a comprehensive psychiatric evaluation of the patients, blinded to the crisis triage rating. Diagnoses were based on unstructured clinical interview, according to the ICD-10 criteria, as part of routine clinical assessment. Data were entered into an electronic database.

### 2.4. Measures

#### 2.4.1. Crisis Triage Rating Scale (CTRS)

The CTRS [13] was used to rate the level of urgency of all presentations to the PES. CTRS is a 3-item clinician-rated scale that assesses the level of urgency of emotional crisis in psychiatric emergency settings, on the domains of suicidality or dangerousness, support system, and cooperativeness. All the domains are scored on a scale of 1 to 5. The first domain assesses the degree of dangerousness of the patient to self or others (1 = most dangerous, 5 = least dangerous), the capability and willingness of the patient's family or other social support network to assist in the treatment plan (1 = poor support system, 5 = excellent support system), and the patients' motivation and ability to cooperate with an out-patient treatment plan (1 = least motivated, 5 = most motivated). A sum of the three domains provides the crisis triage rating score which could range from 3 (Emergency crisis) to 15 (routine crisis). Based on the CTRS scores, patients are categorised into routine (11 to 15), urgent (9 to 10) and emergency (3 to 8). For ease of presentation, urgent and emergency presentations were merged together as “urgent.” The instrument has been shown to be reliable in our study setting [14]. Interrater reliability on a small subset (*n* = 21) of the current sample was also satisfactory (*r* = 0.91, *P* < 0.001).

#### 2.4.2. Clinical Global Impression Scale

The Clinical Global Impression (CGI) Scale [15] consists of two items, out of which the first item was used in this study. The first item, the “Global Severity” item, requires the clinician to rate the severity of the mental illness in a patient at the time of assessment relative to the past experience of the clinician with patients who have the same diagnosis, on a scale of 1 to 7 (1 = normal, not ill at all, and 7 = extremely ill). This instrument provided a proxy measure of the urgency of mental health problems at presentation. The CGI has been widely used in our study setting. Inter-rater reliability on a small subset of our sample was satisfactory (*r* = −0.82, *P* < 0.001).

#### 2.4.3. Supplementary Questionnaire

The questionnaire designed by the authors consisted of three sections. The first part elicited sociodemographic data such as age, gender, marital status, and employment status. The second section collected data on some service use variables such as the number of visits to the emergency department in the past year and engagement with routine out-patient psychiatric care facility such as out-patient clinics. The latter item assessed whether or not patients had defaulted (dropped out) from attending scheduled routine out-patient appointments for psychiatric care. The third section contained clinical data abstracted from a comprehensive psychiatric evaluation including psychiatric diagnoses according to the ICD-10 diagnostic criteria and the chief complaints (reasons for presentation). Specific diagnoses were made, but only broad categories are presented here.

### 2.5. Statistical Analysis

Data was analysed with SPSS version 16. The primary outcome variable was the level of urgency of mental health problems at presentation to the PES, according to the CTRS scores, dichotomised into urgent and nonurgent categories. Patients with urgent and emergency presentations were collapsed under the “urgent” category. Chi-square test was used to examine the association between the outcome variable and categorical independent variables. To test for independent significant relationships, significant variables on bivariate analysis were entered into logistic regression analysis. The test was essentially two-tailed with level of significance set at *P* < 0.05.

## 3. Results

The sample consisted of 700 patients who consecutively presented to our psychiatric Emergency Unit over a period of three months. The age of the participants ranged from 14 to 69 years, with mean age of 36.8 (±10.9) years (Table 1). Females constituted 59.1% of the sample. About one out of every three (34.6%) participants were married and 39.7% were employed.

Table 2 shows the pattern of CTRS and CGI scores of the participants. Less than a third (29.1%) of the presentations were an “emergency” and 10.9% were “urgent,” while 60% were “nonurgent” based on previously defined CTRS scores. Altogether, only 40% of the patients presenting to the Emergency Unit had “urgent” or “emergency” mental health problems. There was significant correlation between CTRS and CGI scores (*r* = −0.82, *P* < 0.001).

Of the 420 patients who presented with nonurgent problems, 196 (46.7%) needed prescription refill only (Table 3), which was commonly attributed to insufficient funds to buy enough drugs at their last clinic visit. Out of the 280 patients with urgent problems, 66 (23.8%) were mechanically restrained at home by family members before presentation, while 36 (12.9%) were brought by police or ambulance.

The factors associated with the level of urgency of presentations to the Emergency Unit are highlighted in Table 4. Patients with nonurgent problems were more likely to present unaccompanied (*P* < 0.001), have need for prescription writing/medication refill only (*P* < 0.001), be employed (*P* < 0.001), and be married (*P* < 0.001). Urgent presentations were more common among patients with substance abuse (*P* < 0.001), history of suicidal attempt or contemplation (*P* < 0.001), defaulters from routine out-patient clinics (<0.001), patients accompanied by police or ambulance (*P* < 0.001), and those who were mechanically restrained (e.g., ropes) at home prior to presentation (*P* < 0.001).

On regression analysis (Table 5), employment status, need for medication refill only, substance use, suicidality, and mechanical restraint application independently predicted urgency of mental health problems among patients presenting to the emergency department.

## 4. Discussion

To the best of our knowledge, this is the first study of the pattern and correlates of urgency of mental health problems among patients attending a psychiatric emergency service in sub-Saharan Africa. We found that 6 out of every 10 presentations were “nonurgent.” This result supports previous findings that Psychiatric Emergency Services are often used by patients who could have been served by routine mental health services such as out-patient clinics [16–18].

Though methodological differences preclude strict comparability across studies, the rate of utilisation of PES for nonurgent problems in the current study was higher than that reported in western populations [5–9]. Previous authors have shown that the pattern of utilisation of PES is a proxy indicator of the performance of the mental health service, as unmet needs in other components of the health system will lead to an upsurge in use of Emergency Services [4, 19].

The excessive use of PES for nonurgent problems may partly reflect the poor access to routine mental health care in the country [4, 19]. In Nigeria, mental health care is grossly underresourced, payment for treatment is out-of-pocket [20], and the majority of the services are isolated in 8 psychiatric hospitals [21, 22]. There is a very high rate of drop-out from routine out-patient psychiatric clinic due to the long distance from the patients home to the nearest psychiatric care facility (>100 km in many instances) and lack of funds for transportation or payment of hospital/drugs fees [23].

The patients that needed prescription refill alone constituted 46.7% of those with nonurgent presentations. The most common reason for the need to refill medication was due to lack of funds to procure sufficient medications. Clinicians working in emergency departments in resource-poor settings where there are limited routine treatment options may need to exercise caution in quickly dismissing patients as “cold cases” as such overzealous triaging may result in “revolving door” patients. Furthermore, if services not cater, for these patients, their problems may escalate into a crisis, thereby adding to the pool of defaulters who present in emergencies.

Employed patients were more likely to present with nonurgent problems than unemployed patients (*P* < 0.001). This contrasts with findings in western studies. In Nigeria, mental illness is highly stigmatised in the workplace, and there is little or no legislative support for the protection of the rights of employees or to shield employees from being victimised on account of mental illness. Therefore employed patients are likely to conceal their history of mental illness, which may limit their opportunity to attend routine out-patient clinics with hours of operation coinciding with regular working hours. Such patients may prefer to use the PES due to flexibility of hours of operation and lack of necessity for previous scheduled appointment. Another possible interpretation is that patients with more urgent presentations are more likely to be severely ill and consequently have lesser likelihood of functioning occupationally. In addition, they may not be able to retain their jobs due to stigma and discrimination by coworkers or/and their employers. Furthermore, in Nigeria where “payment for services is out-of-pocket” rather than by health insurance cover, unemployed patients may not be able to afford services until symptoms become severe or urgent.

Patients who had dropped out of treatment were more likely to present with urgent problems/emergencies (*P* < 0.001). This is consistent with previous reports that nonengagement with appropriate out-patient treatment correlates with the excessive use of Emergency Services [8, 24]. Furthermore, the result supports the argument from the perspective of secondary prevention that nonurgent presentations may represent an early cry for help with the view of forestalling exacerbation and eventual presentations in crisis [25].

The significant association between abuse of substances and crisis presentation is in agreement with the literature. Patients with substance use problems are overrepresented among emergency room attendees and are likely to be multiple users of services [26, 27]. The abuse of substances may be underrecognised as a problem that warrants psychiatric intervention until onset of acute complications which may deteriorate into a crisis. Substance use is also associated with high rate of default from routine services and high risk of relapse. PES must be equipped to provide comprehensive evaluation of patients with substance abuse related problems and ensure efficient linkages with services for definite treatment and rehabilitation.

Patients with history of suicidal contemplation or attempt were more likely to present in crises (*P* < 0.001). The finding is consistent with previous reports in the literature [25, 28]. Services for suicidal patients presenting to the emergency department must be scaled up to include interventions targeted at engaging suicidal patients in treatment even after discharge from the emergency department [28].

The proportion of patients with urgent problems who were brought in by ambulance or police (12.9%) was lesser than those brought in with physical restraint (23.8%). The low rates of patronage of ambulance services or police involvement may reflect the difficulties involved in accessing these services when patients need them. The inappropriate use of restraints (chains, ropes, etc.) may be attributed to ignorance about the nature of mental illness, negative attitudes, and stigma, and it highlights the need for interventions designed to abate the practice.

Our study had some limitations. The urgency of mental health problems was determined by the CTRS scores. The use of an instrument in rating severity or urgency of mental health problems is not completely foolproof; urgency of presentations could have been underestimated or overestimated. However, the CTRS is a widely used instrument, and has been previously used in our setting. We also found satisfactory inter-rater reliability in the current study and a satisfactory correlation with CGI, another widely used measure. Our results should be considered in the context of the study setting; generalisation to settings with different service structure may be limited. Other complex service-related or user-related variables may be related to the urgency of presentations rather than the variables we investigated. The sample size may also be a limitation. However, this study has provided very salient information on the pattern and correlates of urgency of mental health problems among patients attending psychiatric emergency service in a previously underresearched setting. We used a prospective design rather than a retrospective review of electronic clinical records.

## 5. Conclusion

Six out of ten presentations to Psychiatric Emergency Services in our setting were for nonurgent problems, a rate higher than that reported in western populations. A significant proportion of patients with “nonurgent” problems needed medication refill only. The majority of patients with urgent presentations were defaulters and were more likely to use substance, have suicidal tendencies, be accompanied by police, or be brought in with mechanical restraint. Efforts to ensure appropriate use of services must be directed towards improved education of service users on the appropriate point of care-seeking depending on the nature of the problem and the need for compliance with routine out-patient clinic appointments. Health care financing policy should be revised to support continuous access to medications for patients who cannot afford them. There is also need to scale up access to mental health services in the community. Pending the implementation of the necessary reforms, the Emergency Department may remain the only safety net for patients presenting to it.

## Figures and Tables

**Table 1:** Sociodemographic characteristics of the participants (*N* = 700).

Variable	*n*	%
Age in years (mean ± SD)	36.8 (±10.9)	
Sex		
Male	286	40.86
Female	414	59.14
Marital status		
Married	242	34.57
Not married	458	65.43
Employment status		
Employed	278	39.71
Unemployed	422	60.29
Mode of presentation		
Alone	103	14.71
With relative, no restraint	479	68.43
With relative, in restraint	74	10.57
By police/ambulance	44	6.29

**Table 2:** Pattern of CTRS and CGI scores (*N* = 700).

Variable	*n* (%)
CTRS scores	
3–8 (emergency)	204 (29.14)
9-10 (urgent)	76 (10.86)
11–15 (nonurgent)	420 (60.00)
CGI scores	
6-7	233 (33.29)
4-5	102 (14.57)
1–3	365 (52.14)

CTRS: Crisis Triage Rating Scale.

CGI: Clinical Global Impression Scale.

**Table 3:** Distribution of diagnosis and presenting complaints.

	*N* = 280	*N* = 420
	Urgent	Nonurgent
	*n* (%)	*n* (%)
(ICD-10) Psychiatric diagnoses		
(F00–F09) Organic mental disorder	20 (7.14)	32 (7.62)
(F00–F09) Psychoactive substance abuse	52 (18.57)	28 (6.67)
(F20–F29) Schizophrenia/others	132 (47.14)	196 (46.67)
(F30–F39) Mood disorders	62 (21.14)	108 (25.71)
(F40–F49) Neurotic disorders	1 (0.37)	36 (8.57)
Other ICD-10 disorders	13 (4.64)	20 (4.76)
Presenting complaints*		
Nil (medication refill only)	7 (2.50)	196 (46.67)
Aggressive behaviour	158 (56.43)	—
Suicidality	101 (36.07)	—
Mood/anxiety symptoms	121 (43.21)	187 (44.52)
Delusions/hallucinations	238 (85.0)	169 (39.76)
Substance use	52 (18.57)	28 (6.67)
Insomnia	132 (47.14)	148 (35.24)
Refusal to eat	48 (17.14)	8 (1.91)
Other complaints	163 (58.21)	73 (17.38)

*Total > 100% because of multiple complaints.

**Table 4:** Factors associated with urgency of presentations.

Variable	Urgent *n* (%)	Nonurgent *n* (%)	Total	*χ* ^2^	P
Sex					
Male	118 (41.3)	168 (58.7)	286	0.32	0.572
Female	162 (39.1)	252 (60.9)	414		
Marital status					
Married	70 (28.9)	172 (71.1)	242	18.90	**<0.001**
Not married	210 (45.9)	248 (54.1)	458		
Employment status					
Employed	86 (30.9)	192 (69.1)	278	15.79	**<0.001**
Unemployed	194 (46.0)	228 (54.0)	422		
Physical restraint					
Yes	66 (89.2)	8 (10.8)	74	83.42	**<0.001**
No	214 (34.2)	412 (65.8)	626		
By police/ambulance					
Yes	36 (81.8)	11 (23.4)	44	28.12	**<0.001**
No	244 (37.4)	409 (62.6)	653		
Number of PES visits in the past year					
>2	100 (35.7)	180 (64.3)	280	3.57	0.059
≤2	180 (42.9)	240 (57.1)	420		
Engagement with routine outpatient care					
Defaulted	216 (58.7)	152 (41.3)	368	11.36	**<0.001**
Not defaulted	64 (19.3)	268 (80.7)	332		
Refill drugs					
Yes	7 (3.4)	196 (96.6)	203	15.92	**<0.001**
No	273 (54.9)	224 (45.1)	497		
Substance use					
Yes	52 (65.0)	28 (35.0)	80	23.52	**<0.001**
No	228 (36.8)	392 (63.2)	620		

Bold font refers to the significant *P* values (<0.005).

**Table 5:** Regression analysis of factors associated with urgency of presentation.

Variable	*B*	SE	Wald	*P*	OR	95% CI
Employment status	−0.48	0.23	4.49	0.034	0.62	0.39–0.43
Marital status	−0.21	0.21	0.95	0.329	0.81	0.52–0.73
Physical restraint	2.47	0.43	33.11	<0.001	11.79	5.01–7.61
With ambulance/police	0.33	0.38	0.77	0.381	1.39	0.61–0.78
Defaulting	0.95	0.22	19.06	<0.001	2.59	1.64–1.92
Need to refill drugs	−2.79	0.41	45.98	<0.001	0.06	0.07–0.09
Suicidality	1.26	0.34	17.32	<0.001	5.42	1.86–3.52
Substance use	1.15	0.30	14.34	<0.001	3.16	1.74–2.91

OR: odds ratio.
